# Thyroid–Microbiome Allostasis and Mitochondrial Performance: An Integrative Perspective in Exercise Physiology

**DOI:** 10.3390/nu18010059

**Published:** 2025-12-24

**Authors:** Adrian Odriozola, Adriana González, Iñaki Odriozola, Francesc Corbi, Jesús Álvarez-Herms

**Affiliations:** 1 Department of Genetics, Physical Anthropology and Animal Physiology, University of the Basque Country (UPV/EHU), 48940 Leioa, Spain; adriana.gonzalez@ehu.eus; 2 Institut Nacional d’Educació Física de Catalunya (INEFC), Centre de Lleida, Universitat de Lleida (UdL), 25003 Lleida, Spain; jesusah80@gmail.com; 3 Health Department of Basque Government, 20013 San Sebastian, Spain; iongideon@hotmail.com; 4 Department of Clinical Sciences, Faculty of Medicine and Health Sciences, University of Barcelona, 08907 L’Hospitalet de Llobregat, Spain; f@corbi.neoma.org; 5 Phymo^®^ Lab, Physiology and Molecular Laboratory, 40170 Collado Hermoso, Spain

**Keywords:** thyroid hormones, gut microbiota, mitochondria, exercise physiology, allostasis, short-chain fatty acids, deiodinases, micronutrients, adaptive fatigue, precision nutrition

## Abstract

Exercise acts as a physiological stimulus, requiring precise coordination among endocrine, microbial, and mitochondrial systems to maintain metabolic stability through allostatic regulation. The goal of the article is to integrate multidisciplinary evidence to characterize the thyroid–microbiome–mitochondrial axis as a key regulator of the allostatic state in athletic physiological response. During acute, chronic, and overload training phases, the thyroid–microbiome–mitochondrial axis operates bidirectionally, coupling microbial signaling with endocrine and mitochondrial networks to mediate metabolic response to exercise. This response shows interindividual variability driven by sex, age, genetics, and nutritional status, shaping the boundaries between adaptive efficiency and allostatic overload. Microbial metabolites, such as short-chain fatty acids (SCFA) and secondary bile acids, modulate deiodinase activity, bile acid recycling, and mitochondrial biogenesis through AMPK–SIRT1–PGC1α signaling, optimizing substrate use and thermogenic capacity. Thyroid hormones reciprocally regulate gut motility, luminal pH, and bile secretion, maintaining microbial diversity and mineral absorption. Under excessive training load, caloric restriction, or inadequate recovery, this network becomes transiently unbalanced: SCFA synthesis decreases, D3 activity increases, and a reversible low-T_3_/high-rT_3_ pattern emerges, resembling early Hashimoto- or Graves-like responses. Selenium-, zinc-, and iron-dependent enzymes form the redox link between microbial metabolism, thyroid control, and mitochondrial defense. In conclusion, the thyroid–microbiome–mitochondrial axis provides the physiological basis for the allostatic state, a reversible phase of dynamic recalibration that integrates training, nutrition, environmental stress, and circadian cues to sustain thyroid activity, mitochondrial efficiency, and microbial balance. This integrative perspective supports precision interventions to optimize recovery and performance in athletes.

## 1. Introduction

### 1.1. Exercise as a Model of Endocrine and Metabolic Allostasis

Thyroid hormones (THs) serve as central regulators of metabolic homeostasis, linking energy expenditure, thermogenesis, and cellular adaptation to environmental and physiological demands [1]. Rather than functioning solely as static controllers of basal metabolism, they can act as adaptive modulators of energetic efficiency and tissue remodelling in response to exercise [2].

Through these integrative actions, THs coordinate mitochondrial and immune metabolism and interact with microbial networks that influence substrate utilization and systemic energy efficiency [3]. From an allostatic perspective, thyroid regulation sustains stability by change, continuously recalibrating hormonal output and receptor sensitivity to match the organism’s energetic and environmental context [4].

This adaptive flexibility is crucial in sport physiology, where energy turnover fluctuates across training cycles, nutritional phases, and recovery states. In athletes, transient alterations in circulating THs reflect regulatory adjustments rather than dysfunction, mirroring the balance between metabolic demand and available resources [2].

THs exert these effects through both central and peripheral integration. Systemically, they coordinate with the hypothalamic–pituitary–thyroid (HPT) axis to maintain endocrine balance. In contrast, at the tissue level, they modulate mitochondrial biogenesis, oxidative phosphorylation, and thermogenic efficiency. These multi-tiered actions enable the body to switch between energy mobilization and energy-sparing modes according to workload, temperature, and nutritional state [3,5,6].

Importantly, thyroid-driven metabolic regulation interacts with other physiological systems heavily engaged during exercise, including skeletal muscle, cardiovascular, and immune pathways. By modulating oxygen consumption, substrate oxidation, and redox balance, THs fine-tune performance, recovery, and adaptation. When this adaptive mechanism is chronically challenged by overtraining, energy deficit, or environmental extremes, regulatory flexibility may become constrained [2,7].

### 1.2. Exercise as an Endocrine Stressor

Physical exercise is one of the most potent systemic stressors on the endocrine network, requiring coordinated adjustments in energy turnover, substrate availability, and thermoregulation [8]. The thyroid system plays a pivotal role in this adaptive process, continuously recalibrating its hormonal output and tissue responsiveness to sustain metabolic efficiency under fluctuating energetic conditions [2].

From an allostatic perspective, exercise challenges the stability of the HPT axis, eliciting transient deviations in hormone levels that serve as signals of adaptation rather than dysfunction [4,9].

Acute exercise induces rapid and intensity-dependent endocrine shifts. Moderate workloads generally increase circulating thyrotropin (TSH) and free T_4_ (fT_4_), facilitating substrate mobilization and oxygen consumption [10,11,12,13].

In contrast, prolonged or high-intensity efforts may transiently reduce triiodothyronine (T_3_) and enhance the production of reverse T_3_ (rT_3_), a metabolic signal associated with reduced energetic turnover [14,15,16,17]. These acute hormonal inflections exemplify the allostatic principle of *stability through change*, reflecting the body’s capacity to fine-tune thyroid activity according to energetic demand.

Across training cycles, chronic exercise acts as a regulatory force, shaping endocrine set points. Population studies and controlled interventions indicate that habitual physical activity is associated with lower basal TSH and slightly higher fT_4_, patterns compatible with improved metabolic efficiency and mitochondrial coupling [14,18,19]. In well-adapted athletes, this recalibration supports enhanced oxidative metabolism and faster recovery. However, when training loads exceed recovery capacity or are compounded by caloric restriction, circadian disruption, or environmental stress, the same regulatory mechanisms can shift toward **allostatic** overload. This maladaptive state resembles the biochemical profile of the non-thyroidal illness syndrome (NTIS). NTIS is characterized by reduced T_3_, suppressed TSH, and elevated rT_3_ and is often accompanied by fatigue, impaired thermoregulation, and slower recovery [8,20,21,22].

Environmental and behavioral modulators further shape these endocrine dynamics. Sleep restriction, misaligned feeding schedules, and circadian rhythm disruption alter thyroid hormone pulsatility and feedback sensitivity [19,22]. Similarly, repeated exposure to heat, cold, or hypoxia amplifies endocrine stress, modifying the kinetics of hormone production and clearance [23,24]. When superimposed on high training volumes, these contextual factors determine whether thyroid regulation enhances resilience or drifts toward suppression.

A systematic review and meta-analysis of randomized controlled trials involving adults with subclinical hypothyroidism showed that exercise-based interventions of ≥12 weeks led to a significant decrease in serum TSH and an increase in free T_4_ [18]. These data, derived from randomized controlled trials, provide the strongest population-level evidence to date that long-term structured exercise produces adaptive recalibration of the HPT axis rather than pathological suppression. In this review, adaptive recalibration refers to the reversible adjustment of hormone availability and tissue sensitivity that enhances metabolic efficiency under repeated training exposure, consolidating the clinical dimension of thyroid allostasis.

### 1.3. The Gut–Thyroid Axis: Bidirectional Regulation and Dysbiosis-Related Phenotypes

The gut–thyroid axis constitutes a bidirectional communication network linking microbial ecology, endocrine signaling, and immune regulation. This interaction becomes especially relevant under fluctuating metabolic and environmental demands, where intestinal and thyroid regulation are tightly interconnected. The resulting cross-talk determines whether energy metabolism and recovery proceed efficiently or the system drifts toward maladaptation under sustained stress.

**Figure 1:**
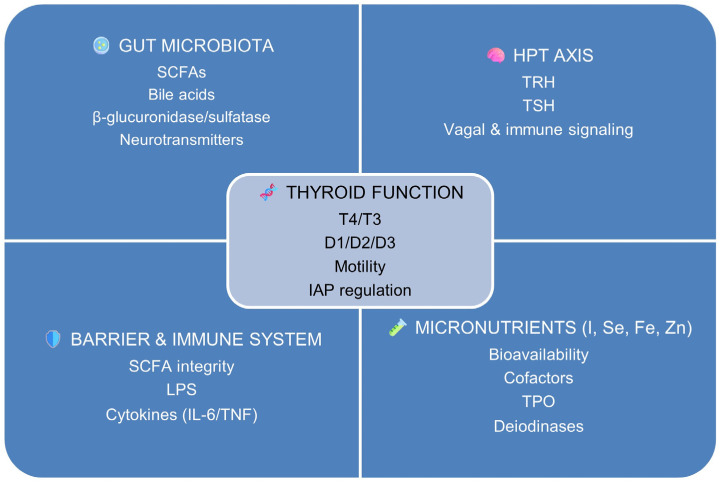
Schematic representation of the core domains composing the gut–thyroid axis. Thyroid function (center) acts as an integrative hub linking four interconnected regulatory systems: the gut microbiota (producer of short-chain fatty acids, SCFAs; bile acids; and microbial enzymes such as β-glucuronidase/sulfatase), the hypothalamic–pituitary–thyroid (HPT) axis [thyrotropin-releasing hormone (TRH) and thyroid-stimulating hormone (TSH)], micronutrient-dependent pathways (iodine, I; selenium, Se; iron, Fe; zinc, Zn), and the intestinal barrier–immune interface [lipopolysaccharide (LPS) exposure and pro-inflammatory cytokines, including interleukin-6 (IL-6) and tumour necrosis factor-α (TNF-α)].

Figure 1 outlines the major regulatory domains: microbial, endocrine, immune–barrier, and micronutrient-dependent, that collectively sustain or disrupt thyroid homeostasis under training-related stress.

These domains converge to modulate thyroid hormone availability (thyroxine, T4; triiodothyronine, T3), peripheral activation and inactivation via deiodinases (D1, D2, D3), gastrointestinal motility, and epithelial regulation of intestinal alkaline phosphatase (IAP). The figure provides a conceptual and physiological framework for the gut–thyroid axis described in Section 1.3, rather than a quantitative or experimental model.

Dysbiosis is a quantitative, qualitative, or functional alteration of the intestinal microbiota that typically involves the loss of microbial diversity, depletion of short-chain fatty acid (SCFA) producing taxa such as *Faecalibacterium* and *Roseburia*, and enrichment of mucin-degrading or pro-inflammatory bacteria such as *Ruminococcus torques* and *Enterobacteriaceae* [25,26]. Such alterations influence intestinal barrier status, bile acid dynamics, and micronutrient handling, with downstream effects on endocrine regulation. The result is a disturbance in the delicate balance that normally sustains endocrine and metabolic efficiency, particularly during intense training or insufficient recovery [27].

The intestinal microbiota influences thyroid function through several interdependent mechanisms. It modulates the absorption and bioavailability of key micronutrients, notably iodine, selenium, iron, and zinc, which act as essential cofactors for thyroid peroxidase (TPO) and deiodinase activity [28,29,30]. Commensal bacteria such as *Lactobacillus* and *Bifidobacterium* enhance mineral solubility and epithelial uptake, whereas dysbiosis limits their availability and compromises hormone synthesis and activation.

Microbial hydrolases, including β-glucuronidases and sulfatases, contribute to the enterohepatic recycling of THs by deconjugating iodothyronine metabolites such as T_4_ and T_3_ during intestinal passage [23,31,32,33]. In parallel, bacterial metabolism of bile acids affects the solubility and reabsorption of iodothyronines, further linking microbial composition to thyroid hormone kinetics [34,35,36,37]. Dysbiosis that disrupts these processes can therefore alter the balance between hormone activation (via deiodinase types 1 and 2, D1/D2) and inactivation (via type 3, D3).

Microbial signaling extends to the central neuroendocrine axis through immune and vagal pathways. Gut bacteria are capable of producing or modulating neurotransmitters such as dopamine, serotonin, and γ-aminobutyric acid (GABA), which can influence vagal afferents and hypothalamic circuits involved in neuroendocrine regulation. This interaction provides a plausible mechanism through which the gut microbiota may indirectly modulate TSH-releasing hormone (TRH) and TSH secretion, although direct experimental evidence remains limited [38,39,40].

Exercise-induced changes in dopaminergic and serotonergic tone influence hypothalamic regulation of stress and energy balance [41,42]. Microbially derived SCFA from fibre fermentation promote regulatory T-cell differentiation and anti-inflammatory signalling [26,43,44]. In athletic dietary contexts characterized by low fibre and high protein, reduced SCFA production may weaken immunoregulatory defences, potentially facilitating immune dysregulation. Conversely, THs regulate gastrointestinal ecology, influencing motility, epithelial turnover, and enzyme expression [45,46]. Hypothyroidism is associated with reduced transit and possible bacterial overgrowth, whereas hyperthyroidism may accelerate transit and alter microbial communities [47]. Impaired thyroid signalling may reduce intestinal alkaline phosphatase (IAP) activity, potentially promoting endotoxemia-related immune activation, a mechanism plausible in athletes under metabolic stress but not fully established.

### 1.4. Knowledge Gaps and Objectives of the Review

Despite significant progress in elucidating the interplay between thyroid function, gut microbiota, and exercise adaptation, current knowledge remains fragmented and methodologically inconsistent. Existing studies differ markedly in the population type, analytical resolution, and control for confounding factors such as diet, training load, or circadian rhythm. These disparities foster inconsistencies and limit translational potential [48,49]. Most investigations rely on small or cross-sectional cohorts, which hinder distinguishing adaptive physiological fluctuations from pathological alterations and obscure the temporal dynamics of thyroid–microbiota interactions under athletic stress.

A key limitation lies in the isolation of endocrine, microbial, and secondarily genetic components, which are seldom analysed as elements of a unified regulatory network. The Thr92Ala polymorphism of DIO2 (rs225014) has been associated with reduced deiodinase efficiency and mitochondrial alterations [50,51]. The roles of additional variants such as DIO1, THRB, and TSHR remain poorly characterised, particularly in athletic populations [52]. Other loci involved in mitochondrial biogenesis, oxidative balance, and immune modulation, such as PGC1A, NRF1/2, SOD2, and HLA, may interact with microbial metabolites and nutrient fluxes, shaping thyroid allostasis and inter-individual performance responses. Yet truly integrative analyses combining these layers are still exceptional [48,49].

Nutritional and environmental determinants further complicate this landscape. Imbalances in iodine, selenium, iron, or zinc influence thyroid hormone synthesis and activation. At the same time, dietary fibre and polyphenols modulate SCFA production, bile acid metabolism, and intestinal permeability, factors that, in turn, feed back into thyroid regulation [27,53]. Circadian disruption, caloric restriction, and environmental stressors such as heat, cold, or hypoxia also alter endocrine set-points, yet few studies rigorously control for these variables. Consequently, current evidence often presents static snapshots of what is inherently a dynamic, context-dependent system.

Methodological limitations remain equally critical. Most research still relies on linear associations rather than mechanistic or causal frameworks. Multi-omics designs integrating genomics, metabolomics, metagenomics, and endocrine profiling, using approaches such as Mendelian randomisation or network-based modelling, are scarce. Without these tools, progress from descriptive to predictive and individualised models of thyroid regulation remains elusive [48,49].

This review follows a mechanism-oriented approach. Studies were included based on their relevance to thyroid regulation, mitochondrial physiology, gut microbial function, and exercise-induced metabolic stress. Evidence was drawn from human athletic cohorts, clinical populations, controlled trials, and mechanistic studies in animal and cellular models. Human findings were prioritized when available, while preclinical data were used to contextualize pathways not yet demonstrated in athletes. Studies were excluded when they did not address thyroid regulation, mitochondrial function, gut microbial activity, or their modulation by exercise, or when the mechanistic connection to the review’s scope was insufficient.

Against this background, the present review seeks to synthesise and critically evaluate existing evidence on the exercise–gut microbiota–thyroid triad from an allostatic and integrative perspective. Specifically, we aim to: (I) delineate the endocrine and molecular bases of thyroid adaptation to physical stress; (II) examine how microbial metabolites and micronutrient dynamics influence hormone synthesis, activation, and signalling in athletes; and (III) outline interdisciplinary frameworks for monitoring and optimising thyroid function in sport. By situating thyroid allostasis within the broader context of host–microbiome integration and performance physiology, this review aims to contribute to the transition from associative paradigms toward more predictive and mechanistic frameworks.

## 2. Mechanistic Foundations of Thyroid Allostasis in Exercise Physiology

### 2.1. The HPT Axis, Hormonal Thresholds, and Interindividual Variability in Thyroid Allostasis

The HPT axis constitutes a dynamic regulatory network that continuously calibrates hormonal output and tissue sensitivity to sustain energy balance in response to changing physiological demands. Rather than maintaining static homeostasis, it operates allostatically, redefining its set points in response to physical, nutritional, and environmental challenges [4,9,54]. In athletes, this flexible recalibration determines the boundary between endocrine resilience and maladaptation, dictating whether training stress enhances performance or precipitates fatigue and impaired recovery [55].

At the central level, TRH secreted by the hypothalamic paraventricular nucleus stimulates pituitary release of TSH, which in turn drives thyroidal secretion of thyroxine (T_4_) and T_3_ [24,56]. Circulating T_3_ and T_4_ exert negative feedback on both the hypothalamus and the pituitary, maintaining a dynamic equilibrium [1]. However, exercise, caloric restriction, circadian misalignment, or thermal stress can transiently alter this sensitivity, producing fluctuations in TSH or free hormone levels that reflect adaptive recalibration rather than dysfunction [15,17,21,22].

In plasma, over 99% of THs circulate bound to carrier proteins, while less than 1% remains free as the biologically active fractions (fT_4_ and fT_3_) [2]. This buffering system provides both stability and responsiveness: bound hormones serve as a reservoir, whereas free hormones enable rapid metabolic adjustments. T_4_, with a half-life of roughly seven days, functions primarily as a prohormone, while T_3_, with a half-life of approximately one day, acts as the principal effector, enhancing mitochondrial respiration, oxygen consumption, and thermogenesis [57]. Under metabolic stress, inner-ring deiodination produces rT_3_ and reverse T_4_ (rT_4_), which inhibit thyroid-receptor activity and dampen mitochondrial flux to conserve energy [16]. During intense or prolonged exercise, transient elevations in rT_3_/rT_4_ have been observed in several cohorts and may represent an adaptive endocrine response to oxidative overload [14,18]. Persistent elevation, however, indicates impaired recovery or excessive allostatic load.

Tissue-specific regulation depends on local deiodinase activity, which finely tunes the activation and inactivation of THs. D1 and D2 catalyse outer-ring deiodination, converting T_4_ to active T_3_, whereas D3 catalyses inner-ring deiodination, generating inactive metabolites (rT_3_, T_2_) [57]. D1 predominates in the liver and kidney, supplying systemic T_3_. D2 is abundant in skeletal muscle, brain, pituitary, and brown adipose tissue, allowing swift local adjustments to energy demand [58,59,60]. D3 acts as a metabolic brake, up-regulated during stress, caloric restriction, or inflammation to limit energy expenditure [16,61].

Physical exercise exemplifies this adaptive spectrum. Moderate endurance training typically elevates fT_4_ and maintains or slightly increases fT_3_ while lowering basal TSH, consistent with improved metabolic efficiency and tighter feedback control [10,14,18]. When workload exceeds recovery capacity, fT_3_ declines and rT_3_ rises, indicating D3-mediated inactivation and ATP conservation [21,22,59]. This reversible pattern, resembling NTIS, reflects an adaptive allostatic suppression rather than pathology [62]. Prolonged suppression, however, signals a shift beyond the adaptive threshold, manifesting as chronic fatigue, reduced thermogenesis, and delayed recovery [20].

Considerable interindividual variability characterises these endocrine responses. Genetic polymorphisms modulate the efficiency and sensitivity of thyroid regulation. The DIO2 Thr92Ala variant reduces enzymatic stability and T_4_ to T_3_ conversion, potentially contributing to lower intracellular T_3_ and slower recovery [63,64]. Variants in DIO1, THRB, and TSHR further influence receptor affinity and feedback thresholds [52]. Additional loci, such as PGC1A, NRF1/2, and SOD2, involved in mitochondrial biogenesis and redox control, are likely to interact with thyroid signalling to modulate energy efficiency and fatigue resistance. These differences may partly account for why athletes exposed to similar training loads exhibit divergent thyroid responses, with some maintaining adaptive equilibrium while others develop transient biochemical suppression [20,21].

Such variability could help define allostatic thresholds, the physiological limits within which thyroid adaptation remains beneficial. Below these limits, exercise induces efficient recalibration, characterised by stable fT_3_, adequate fT_4_, and low TSH. Beyond them, cumulative stress or energy deficit leads to sustained elevation of rT_3_/rT_4_, central suppression, and metabolic downregulation. Environmental stressors, including heat, cold, or hypoxia, can further shift these thresholds by altering hormone clearance and peripheral conversion [24,65].

Longitudinal monitoring of fT_3_, fT_4_, rT_3_, and TSH provides a sensitive window into individual thyroid adaptation. Interpreting these indices against personalised baselines distinguishes physiological recalibration from early maladaptation. A disproportionate rise in rT_3_ relative to fT_3_, or a transiently depressed TSH–fT_4_ axis, reflects adaptive central suppression under high allostatic load [14,18,21]. Integrating these endocrine variables with training metrics, recovery dynamics, and microbial profiles may elucidate how systemic and tissue-specific mechanisms interact to sustain endurance, thermogenesis, and resilience, thereby guiding precision recovery strategies in athletes.

### 2.2. Mitochondrial Translation of Thyroid Allostasis: Bioenergetic and Redox Adaptation

The cellular translation of thyroid allostasis occurs primarily within mitochondria, where THs orchestrate energy transduction, oxidative balance, and thermogenic efficiency. By fine-tuning mitochondrial output to match metabolic demand, these hormones link endocrine flexibility with cellular resilience, a coupling that constitutes the physiological foundation of performance adaptation and fatigue resistance in athletes [2,5,16].

T_3_ and its derivatives are key modulators of mitochondrial biogenesis and function. By binding to nuclear thyroid hormone receptors (THRs) and interacting with co-activators such as PGC-1α, T_3_ stimulates transcription of genes involved in oxidative phosphorylation and mitochondrial content [66]. This cascade also activates nuclear respiratory factors (NRF1 and NRF2) and mitochondrial transcription factor A (TFAM), which coordinate the replication and maintenance of mitochondrial DNA and the assembly of respiratory complexes [67]. Collectively, these mechanisms enhance ATP production capacity and substrate flexibility, which are essential for sustained performance during endurance exercise [57].

Beyond its nuclear actions, T_3_ rapidly modulates mitochondrial membranes, stimulating adenine nucleotide translocase and cytochrome c oxidase to increase respiratory flux and proton leak. The metabolite 3,5-diiodo-L-thyronine (T_2_) also enhances mitochondrial respiration and fatty-acid oxidation, as shown in animal and ex vivo models [68,69]. These fast actions provide the cellular analogue of the systemic allostatic response, enabling minute-scale adaptation of ATP synthesis and thermogenesis.

The thyroid–mitochondrial dialogue also engages energy-sensing pathways such as AMP-activated protein kinase (AMPK) and sirtuin-1 (SIRT1), both of which act as molecular translators between energetic stress and nuclear transcription [5]. T_3_-driven AMPK activation promotes β-oxidation, while SIRT1-mediated deacetylation of PGC-1α amplifies mitochondrial renewal and antioxidant defence [70,71,72]. This integrated network supports efficient recovery and resilience against chronic fatigue in athletes.

Redox control is a pivotal component of thyroid-mediated mitochondrial adaptation. T_3_ up-regulates antioxidant enzymes such as superoxide dismutase 2 (SOD2), glutathione peroxidase (GPx), and catalase, maintaining redox homeostasis during increased oxidative flux [73]. Below the allostatic threshold, ROS act as adaptive signals; beyond it, excessive thyroid activation or overtraining induces hyperpolarization, oxidative stress, and functional decline.

THs also modulate thermogenic and uncoupling mechanisms by regulating uncoupling proteins (UCPs), particularly UCP2 and UCP3, in skeletal muscle and brown adipose tissue [68]. Controlled uncoupling enhances metabolic flexibility, whereas chronic overactivation leads to energy inefficiency and fatigue. This coupling–uncoupling equilibrium mirrors endocrine allostasis, balancing performance and protection.

The thyroid–mitochondrial axis, therefore, represents the reciprocal integration of hormonal and cellular adaptations. Endocrine recalibration defines systemic conditions for mitochondrial efficiency, while mitochondrial feedback through ROS and AMP/ATP ratios tunes the HPT axis and deiodinase activity. Disruption of this reciprocity by chronic stress, undernutrition, or inflammation shifts the system toward conservation, suppressing T_3_ signalling and mitochondrial biogenesis, a state shared by hypothyroidism and overtraining [20].

**Figure 2:**
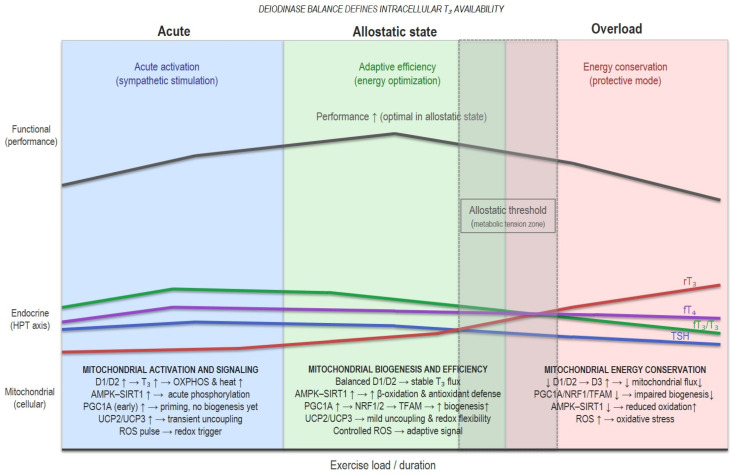
Conceptual model of thyroid–mitochondrial coupling and adaptive thresholds. This figure presents a conceptual model based on an integrative interpretation of the experimental and clinical literature, not on direct measurements. It illustrates how shifts in deiodinase activity, mitochondrial signaling, and redox balance may, in theory, define adaptive versus maladaptive responses to training stress.

Figure 2 shows an integrative model of thyroid–mitochondrial coupling that links findings from cellular, animal, and human studies. Systemic thyroid signals (T_4_, T_3_) are modulated by the balance between activating (D1/D2) and inactivating (D3) deiodinases, shaping intracellular T_3_ availability. In this model, the deiodinase balance defines the endocrine input translated into mitochondrial function. Under adaptive conditions, T_3_ activates PGC1A, NRF1/2, and TFAM, promoting mitochondrial biogenesis and oxidative phosphorylation, while the AMPK–SIRT1 feedback loop enhances fatty acid oxidation and antioxidant response. Controlled ROS generation and mild uncoupling via UCP2/UCP3 contribute to thermogenic and redox flexibility.

When training stress, energy deficit, or sleep loss exceeds recovery capacity, D3 expression increases and D1/D2 activity declines, leading to a state of relative intracellular hypothyroidism. This theoretical transition suppresses PGC1A–NRF1/2–TFAM signalling, and reduces mitochondrial renewal, and oxidative capacity.

The model illustrates this continuum, from acute stimuli to allostatic adaptive recalibration, and eventually to maladaptive suppression in overload, highlighting how shifts in deiodinase dominance (D1/D2 → D3) may shape mitochondrial resilience, oxidative stress, and performance outcomes in athletes. Allostatic threshold refers to the transient zone between adaptive recalibration and maladaptive suppression in overload. This model is hypothetical and intended to synthesize existing mechanisms rather than represent direct empirical measurements.

### 2.3. Thyroid Allostasis in Exercise Physiology: Adaptive Recalibration and Energetic Efficiency

Thyroid responses to exercise operate across distinct temporal scales, from rapid hormonal shifts that accompany acute effort to slower regulatory adjustments that emerge only after repeated training exposure. The following discussion distinguishes these complementary time domains to clarify their physiological significance.

Physical training as a recurrent energetic challenge continuously tests the thyroid system’s ability to preserve homeodynamic balance. Within physiological limits, exercise functions as a controlled stressor, eliciting transient fluctuations in hormone secretion and metabolism that promote adaptive recalibration rather than pathological disruption. These adjustments enhance energy efficiency, tissue remodelling, and recovery, illustrating the principle of stability through change that underlies thyroid allostasis in human performance [2,54].

#### 2.3.1. Acute Endocrine Responses to Exercise

Single bouts of exercise elicit intensity- and duration-dependent changes in circulating THs. Moderate workloads can transiently elevate TSH and fT_4_ concentrations shortly after activity, reflecting sympathetic activation and increased metabolic turnover [10,11]. During prolonged or high-intensity efforts, a temporary reduction in T_3_ and a rise in rT_3_ are sometimes observed, mediated by workload-dependent modulation of deiodinases D1, D2, and D3 [14,22]. These shifts appear context-specific and reversible, limiting excessive ATP consumption and ROS production while preserving metabolic efficiency [2].

Peripheral conversion of T_4_ to T_3_ is influenced by energy availability and redox state, potentially through AMPK–SIRT1–PGC-1α signalling, maintaining hormonal efficiency under transient energetic strain [5]. Such acute fluctuations exemplify an adaptive feedback mechanism: transient T_3_ suppression may prevent mitochondrial overload and ROS accumulation, whereas the subsequent rebound in TSH and fT_4_ during recovery facilitates anabolic processes and tissue restoration. This biphasic pattern has been reported across endurance and resistance modalities, though the magnitude and timing vary with training status, nutrition, and recovery [2,18,74].

#### 2.3.2. Chronic Adaptations to Training Load

Unlike the transient endocrine fluctuations associated with acute physical activity, chronic adaptations arise over repeated training cycles and reflect long-term recalibration of the HPT axis and its metabolic targets. Habitual physical activity is often associated with slightly lower basal TSH and stable or modestly elevated fT_4_, suggesting enhanced peripheral sensitivity and reduced central drive [2,14,18]. This endocrine economy parallels mitochondrial and metabolic adaptations, including greater oxidative capacity, improved fatty acid oxidation, and enhanced antioxidant balance. Elevated PGC-1α, NRF1/2, and SIRT1 expression supports mitochondrial biogenesis and antioxidant defence, while AMPK activation promotes substrate flexibility and fatigue resistance [5,16]. Together, these pathways lower the energetic cost of contraction and reduce lactate accumulation, enabling higher mechanical output for a given hormonal input [75,76].

However, evidence in humans remains heterogeneous. Long-term interventions and meta-analyses in clinical and athletic populations show trends toward reduced TSH and maintained fT_4_, consistent with endocrine efficiency rather than suppression, but results vary depending on sex, nutrition, and training intensity [2,14,18].

At the tissue level, THs facilitate myogenic differentiation, satellite-cell activation, and mitochondrial proliferation, processes essential for long-term adaptation [68]. Adequate thyroid signalling supports hepatic gluconeogenesis, lipolysis, and glycogen restoration, ensuring substrate availability during and after exercise.

These adjustments highlight the integrative role of THs as biochemical mediators between cellular energetics and systemic performance [77,78].

#### 2.3.3. Type and Intensity of Exercise

Training modality and intensity strongly modulate the direction and magnitude of thyroid responses. The patterns described below reflect how different exercise stimuli, from endurance to resistance, interval-based, and to prolonged high-effort formats, impose distinct energetic and inflammatory demands on the HPT axis.

Endurance activities involving sustained aerobic output tend to reduce fT_3_ and raise rT_3_ when energy turnover exceeds recovery capacity [14,22]. In contrast, resistance or high-intensity interval training (HIIT) can elicit transient increases in fT_3_ and TSH immediately post-exercise, reflecting acute sympathetic activation and enhanced substrate turnover [2].

The magnitude of these fluctuations depends on workload, recovery time, and energetic context. Shorter rest intervals or accumulated fatigue narrow the adaptive window, favouring suppression, whereas periodized programs alternating high- and low-intensity sessions facilitate hormonal recalibration and prevent chronic down-regulation [20,21]. Thus, thyroid flexibility mirrors the broader principle of load management: stress drives adaptation only when interspersed with recovery [3,79].

#### 2.3.4. Transition from Performance Gain to Energetic Strain

The capacity for recovery delineates the boundary between adaptive and maladaptive thyroid responses. When training volume, energy deficit, or sleep loss exceeds compensatory capacity, the same mechanisms that enhance efficiency shift toward conservation [9].

Studies in military personnel, ultra-endurance athletes, and overreaching models show declines in fT_3_, increases in rT_3_, and transient TSH suppression following sustained exertion [14,21,22,80]. This endocrine signature reflects early allostatic overload, suggesting that the system has reached a threshold beyond which further stimulation impairs recovery instead of improving adaptation.

Sleep restriction exacerbates this state by blunting nocturnal TSH amplitude and reducing the rhythmicity of thyroid secretion [81,82]. A recent controlled trial demonstrated that even mild sleep curtailment over six weeks significantly reduced nocturnal TSH amplitude and altered fT_4_ dynamics, reinforcing the link between sleep deprivation and HPT-axis dysregulation [83].

Collectively, these findings support a model in which thyroid allostasis maintains metabolic performance under moderate stress but transitions to energy conservation when recovery is insufficient.

### 2.4. Allostatic Overload and Maladaptive Suppression: Endocrine Markers of Fatigue

When cumulative training stress exceeds the system’s adaptive range, thyroid regulation shifts out of its usual flexible operating mode and enters a state characterized by constrained hormonal responsiveness. This transition marks the conceptual boundary between adaptive recalibration and allostatic overload. Overload states involve a set of cellular mechanisms that converge on reduced T_3_ availability and impaired mitochondrial function. These mechanisms arise when energetic demand, inflammatory stress, or insufficient recovery exceed the regulatory capacity of the HPT axis, and thyroid regulation shifts from optimization to protection. In addition to reduced T_3_ availability allostatic overload is characterized by increased rT_3_, and transient TSH suppression a reversible endocrine configuration reflecting functional rather than pathological downregulation [62].

#### 2.4.1. From Adaptive Efficiency to Energy Conservation

During balanced training, transient reductions in fT_3_ or increases in rT_3_ limit oxidative cost without significantly impairing performance. This short-term protective shift becomes chronic suppression when cumulative load persists without adequate recovery or nutrition. Basal TSH tends to decline or remain low-normal; fT_3_ decreases; fT_4_ remains normal or slightly elevated; and rT_3_ rises, a profile indicating peripheral resistance with partial central down-regulation [21,22]. The fT_3_/fT_4_ ratio falls, denoting reduced peripheral conversion and limited intracellular T_3_ availability. In parallel, elevated cortisol and catecholamines reinforce catabolic signalling and further blunt TSH release [15,20].

This endocrine configuration lowers thermogenesis and substrate oxidation to conserve energy. While protective in the short term, its persistence leads to features associated with overtraining, such as fatigue, impaired muscle recovery, and reduced mitochondrial efficiency [5,21]. The transition threshold varies among individuals but typically coincides with chronic energy deficit, sleep loss, or excessive training density [9,14].

#### 2.4.2. Cellular Mechanisms of Overload

Energetic and nutritional status critically shape thyroid responses to training stress. When caloric intake, macronutrient balance, or micronutrient availability fall short of metabolic demand, the HPT axis shifts toward a low-T_3_, energy-conserving profile that amplifies the physiological features of overload.

At the cellular level, thyroid-driven regulation of energy expenditure depends on the balance among deiodinases D1, D2, and D3. Under sustained stress, D3 expression increases in skeletal muscle, liver, and immune tissues, inactivating T_3_ to rT_3_ and T_4_ to rT_4_, while D1/D2 activity decreases [16,57]. The resulting intracellular hypothyroidism suppresses the transcription of mitochondrial biogenic factors, including PGC1A, NRF1, and TFAM, thereby dampening oxidative capacity.

Mitochondrial coupling efficiency declines as AMPK and SIRT1 signalling weaken [5,71]. Reduced stimulation of UCP2 and UCP3 diminishes mild uncoupling, lowering adaptive thermogenesis but increasing electron-transport pressure and ROS generation [73]. Excess ROS oxidizes mitochondrial proteins and lipids, impairing ATP synthase and aggravating fatigue. These alterations transform the high-flux, efficient metabolic phenotype of adaptation into a low-flux, repair-oriented state dominated by stress-response signalling [20]).

#### 2.4.3. Physiological Correlates and Performance Outcomes

The physiological correlates of thyroid downregulation directly influence athletic performance. Alterations in T_3_ availability, mitochondrial efficiency, redox balance, and inflammatory load influence substrate use, endurance capacity, and the ability to sustain or recover from training stress.

Endocrine suppression during overload manifests as multisystem fatigue. Muscularly, it produces lower power output, delayed recovery, and reduced contraction velocity. Cardiovascularly, the resting heart rate rises while heart rate variability declines, reflecting an imbalance in autonomic tone. Immune correlates include low-grade inflammation and redox imbalance, often associated with decreased SCFA production and gut dysbiosis [26,44].

In athletes, this pattern parallels the Relative Energy Deficiency in Sport (RED-S) phenotype, in which reduced T_3_ serves as both a marker and a mediator of metabolic downregulation. Comparable endocrine profiles occur during prolonged expeditions, military operations, or ultra-endurance events, emphasizing that thyroid suppression is a context-dependent systemic adaptation [7,20,22,84].

#### 2.4.4. Recovery and Reversibility

Crucially, this condition is reversible once energy balance and recovery are restored. Refeeding and sleep normalization reactivate D1/D2 pathways, raise fT_3_, and down-regulate D3 [21]. Within days to weeks, the rT_3_/fT_3_ ratio normalizes, mitochondrial biogenesis resumes, and circadian hormonal rhythms recover. Adequate intake of micronutrients, iodine, selenium, iron, and zinc, facilitates enzymatic restoration of thyroid hormone synthesis and activation [85]. The speed and completeness of reversal vary by genetic background and metabolic resilience [63,64].

This reversible suppression should therefore be viewed not as glandular failure but as a signal of energetic mismatch. Early recognition allows distinction between adaptive conservation and pathological dysfunction, a distinction critical for preventing chronic fatigue and performance decline.

### 2.5. Contextual Determinants of Thyroid Response to Training

Recovery involves the progressive reversal of the low-T_3_ phenotype as energetic status, inflammation, and mitochondrial function normalize. These processes restore deiodinase balance, gut–thyroid signalling, and performance capacity, distinguishing reversible athletic suppression from typical pathological thyroid dysfunction.

Thyroid responses to exercise vary widely according to intrinsic and environmental modifiers that shape endocrine sensitivity and recovery capacity [2,7,20].

Sex, age, energy availability, micronutrient status, and circadian alignment all modulate how the HPT axis recalibrates in response to physical stress.

Interpreting hormonal data within this contextual frame is essential to distinguish adaptive flexibility from maladaptive suppression in athletes.

#### 2.5.1. Sex and Age

Sex hormones exert multilevel influences on thyroid physiology. Oestrogens increase T_4_-binding globulin (TBG), elevating total but not free T_4_ and T_3_, whereas androgens and progesterone tend to favour free fractions and peripheral conversion [86]. Women typically present higher TBG and slightly lower fT_3_/fT_4_ ratios, which may increase vulnerability to thyroid down-regulation during caloric restriction or dense endurance training [7]. Conversely, testosterone modulates thyroid metabolism and receptor sensitivity, partly through hepatic protein synthesis and mitochondrial signalling, potentially buffering transient suppression and improving fatigue resilience [87,88].

Age also conditions endocrine plasticity. Younger athletes exhibit more responsive D2 activity and faster T_3_ turnover, whereas aging blunts D1/D2 efficiency and feedback sensitivity, delaying hormonal normalization after intense exercise [57,89]. These differences highlight the need to interpret thyroid biomarkers within sex- and age-specific reference windows, rather than universal cut-offs [90,91].

#### 2.5.2. Energetic and Nutritional Status

Energy availability is the dominant determinant of thyroid allostasis. Caloric deficit and low carbohydrate intake reduce D1/D2 activity while up-regulating D3, lowering T_3_ production, and increasing rT_3_ accumulation [16]. Diminished insulin and leptin signalling further restricts deiodinase activation, amplifying this suppression [92]. Consequently, chronic under-fuelling is common in endurance and weight-category sports, promoting an NTIS-like profile even at moderate training volumes [7]. Thus, energy availability remains the primary modulator of thyroid recalibration during training, linking nutrient flux and hormonal efficiency to recovery potential.

#### 2.5.3. Environmental and Circadian Modifiers

Environmental stressors reshape thyroid signalling via thermogenic and neuroendocrine pathways. Cold exposure elevates TSH and fT_3_, enhancing thermogenesis by activating brown adipose tissue, whereas heat stress and dehydration suppress output to limit metabolic rate [24,65,93]. Hypoxia during altitude training triggers a biphasic T_3_/T_4_ response, initially elevated, later optimized for metabolic economy [94,95].

Circadian alignment critically modulates the HPT axis. TSH exhibits a nocturnal peak and diurnal trough; sleep loss and misaligned feeding schedules flatten this amplitude and dampen thyroid responsiveness, prolonging recovery [22,96,97]. Maintaining consistent light–dark cycles, meal timing, and sleep duration preserves rhythmic secretion and supports endocrine stability.

Each factor dynamically defines an individual’s thyroid set point during exercise stress. Sex and age determine baseline sensitivity; training type and volume shape acute perturbations; nutrition, energy availability, and environment modulate recovery and recalibration. Integrating these determinants establishes the athlete’s personal allostatic threshold, where adaptation ends and conservation begins.

## 3. The Gut–Thyroid–Mitochondrial Axis: Microbial Modulation of Exercise Metabolism

Many of the pathways described in this section, such as SCFA-mediated AMPK–SIRT1 activation, bile-acid-driven D2 stimulation, and Nrf2-dependent redox control, are well characterized in experimental systems but lack direct confirmation in human intervention studies. Their implications for athletic metabolism should therefore be viewed as mechanistic frameworks supported by indirect or associative human evidence rather than fully validated causal relationships.

### 3.1. Microbial Metabolism and Thyroid–Mitochondrial Bioenergetics

#### 3.1.1. Microbial Metabolites Linking Thyroid Signalling and Mitochondrial Function

Physical exercise continuously challenges metabolic and redox balance. The gut microbiota acts as a metabolic interface, modulating thyroid signalling and mitochondrial function, the two main regulators of energy turnover during training and recovery [2,77]. Together, these systems outline a gut–thyroid–mitochondrial axis in which microbial metabolites are thought to shape endocrine and metabolic responses to nutritional and environmental stimuli [2,77].

From a physiological perspective, the gut microbiota and thyroid hormones converge on mitochondrial regulation. SCFAs, particularly butyrate, acetate, and propionate, strengthen epithelial integrity and activate the AMPK–SIRT1–PGC1α axis in experimental models, enhancing mitochondrial renewal, lipid utilization, and redox stability [26,98]. Within the same circuit, T_3_ amplifies mitochondrial transcription and cytochrome c oxidase activity, while T_4_ provides a systemic reservoir for rapid conversion under metabolic demand [66,99]. Collectively, microbial metabolites and thyroid hormones may contribute mitochondrial efficiency and adaptive energy homeostasis, although their specific influence on athletes’ oxidative performance and redox balance under high-intensity or prolonged exercise remains to be studied.

The microbiota also influences thyroid regulation through bile acid metabolism and enzymatic deconjugation of iodothyronines. Commensal bacteria possessing β-glucuronidase and sulfatase activity regenerate conjugated THs excreted in bile, partially restoring active T_3_ and T_4_, by a mechanism described primarily in preclinical systems [31]. Meanwhile, secondary bile acids activate FXR and TGR5 receptors, promoting D2-dependent thermogenic pathways in experimental models [100]. Interactions provide a plausible route by which gut-derived signals could modulate thyroid hormone conversion and energy efficiency, ensuring that mitochondrial output remains synchronized with training load and nutritional state, although confirmation in humans is still limited.

#### 3.1.2. Microbial Dynamics and Adaptive Stress

The gut microbiota acts as a dynamic regulatory interface, continuously adapting to fluctuations in energy flux, nutrient intake, and training-induced inflammation. A state of eubiosis, characterized by high diversity and dominance of SCFA-producing taxa such as *Faecalibacterium prausnitzii* and *Roseburia* spp., supports epithelial integrity, mineral absorption, and balanced immune signalling, with mechanistic evidence mainly from animal studies and human data still mostly associative [27,101]. Under these conditions, thyroid-related metabolism is thought to operate more efficiently: bile-acid pathways that support lipid utilization are maintained, and experimental data suggest that microbial–endocrine balance may help stabilize deiodinase activity and mitochondrial redox function during exercise.

As workload increases, metabolic stress can alter gut transit time, pH, and oxygen availability, potentially transiently shifting microbial composition toward stress-tolerant taxa—such as certain *Enterobacteriaceae*, and away from SCFA producers. These patterns are mainly supported by animal models and by human studies showing exercise- or heat-related gastrointestinal stress, although taxon-specific responses remain strongly influenced by diet. This transient imbalance can be viewed as an allostatic adjustment rather than a pathological state, in which exercise-related stress may lower SCFA output and modestly enhance LPS/TLR4 signalling, as supported by animal studies and limited human observations. These shifts have been suggested to attenuate thyroid hormone activation and energetically costly pathways, although this remains a theoretical interpretation and has not yet been directly demonstrated in humans [2,20,77].

Improved energy status, sleep, and microbial balance are typically accompanied by higher SCFA output and a shift toward microbial stability. To what extent these changes translate into measurable normalization of deiodinase activity remains unknown, as this mechanism has not yet been confirmed in human intervention studies.

This reversible pattern of eubiosis, transient dysbiosis, and recovery resembles a theoretical form of microbial–endocrine periodization. During this adaptive phase, moderate exercise may help sustain microbial diversity and SCFA production, factors linked to AMPK–SIRT1 activation and metabolic flexibility in experimental models. Whether these interactions meaningfully influence T_4_-to-T_3_ conversion or substrate switching in humans remains to be determined [16,26]. In this context, microbial metabolites may support metabolic regulation through SCFA- and bile-acid–mediated pathways, and potentially influence on human thyroid function.

#### 3.1.3. Inflammatory Drift and Redox–Endocrine Imbalance

When the compensatory mechanisms are persistently challenged by ongoing energy deficits or excessive training loads, microbial and redox balance may deteriorate, contributing to continuous activation of the LPS–TLR4–NF-κB pathway rather than overt inflammation. Such activation can elevate IL-6 and TNF-α levels, cytokines known to suppress D1/D2 and promote D3 activity in non-thyroidal-illness models, although the extent to which this pattern fully manifests in athletes remains uncertain [5,16,77,102]. As selenium- and zinc-dependent antioxidant systems such as GPx and thioredoxin reductase (TrxR) become strained under oxidative stress, the thyroid–microbiota–mitochondria triad may shift toward a pro-inflammatory state [103,104]. This redox–inflammatory interaction may facilitate the transition from adaptive allostasis to early maladaptation, in a potentially reversible stage in which nutritional strategies, recovery, and microbial support could help restore physiological balance [20,27].

Beyond endocrine modulation, microbial metabolites may influence neuroendocrine resilience. SCFAs and tryptophan-derived indoles promote brain-derived neurotrophic factor (BDNF) expression and hypothalamic sensitivity to TRH in experimental models, suggesting a possible route by which microbial diversity contributes to central adaptive responses [38,40]. Sustaining microbial diversity through adequate fibre, micronutrients, circadian alignment, and recovery may help preserve thyroid–mitochondrial coupling, although evidence for direct effects on long-term performance resilience in humans remains limited [2,5,98].

In summary, the gut–thyroid–mitochondrial network can be viewed as a biological barometer of training stress: its flexibility may influence whether exercise favours adaptation or drifts toward fatigue and dysbiosis.

#### 3.1.4. Recovery and Restoration to Eubiosis

Prebiotic fibres play a central role in microbial resilience by supporting SCFA production, epithelial repair, and immune balance. Their restoration during recovery helps re-establish the gut environment that enables normalization of thyroid activation and mitochondrial function.

Once microbial diversity and micronutrient sufficiency are restored, through fermentable fibres, selenium, and omega-3 fatty acids, SCFA production often increases and epithelial integrity may recover, while Nrf2-mediated antioxidant responses have been shown to strengthen in experimental models. Whether these shifts directly influence deiodinase activity or attenuate IL-6/TNF-α signalling in humans remains uncertain, as such mechanisms have not yet been demonstrated in intervention studies [5,98]. This gradual re-alignment of microbial, thyroid, and mitochondrial signals can be understood as a return from a low-T_3_ adaptive state toward a more efficient metabolic profile, reflecting the theoretical reversibility of thyroid allostasis within the gut–endocrine network. Importantly, this interface suggests a model of flexible recalibration rather than dysfunction, helping distinguish adaptive fatigue from true thyroid pathology and positioning the microbiota as a potential contributor to endocrine resilience under training stress.

#### 3.1.5. Adaptive–Autoimmune Patterns Within the Thyroid–Microbiota Axis

Several molecular pathways that mediate reversible thyroid suppression in athletes during periods of high training stress may conceptually overlap with those implicated in autoimmune dysfunction under chronic dysbiosis, although the nature and magnitude of this overlap remain still uncertain. In Hashimoto’s thyroiditis, persistent SCFA depletion and Th17 polarization have been associated with loss of tolerance to thyroglobulin (TG) and TPO, contributing to lymphocytic infiltration and tissue damage [27,105]. Conversely, Graves’ disease results from overstimulation of the TSH receptor by thyroid-stimulating immunoglobulins (TSI), leading to hormonal excess and oxidative damage [106].

In athletes, by contrast, neither immune fixation nor irreversible tissue injury is observed. Instead, the gut–thyroid–mitochondrial interaction may oscillate between two physiological extremes. During hypermetabolic phases, transient elevations in catecholamines and T_3_ support energy mobilization. While recovery is characterized by a low-T_3_ adaptive profile that favours conservation and repair. Both states share the immunometabolic framework described in clinical thyroid disorders, but remain reversible thanks to preserved microbial and endocrine feedback [2,20]. Viewed in this context, the microbiota may act as a regulatory buffer that influences whether repeated training stress leads toward adaptation or toward early immune drift, rather than representing a fixed pathological process.

### 3.2. Micronutrients as Modulators of the Gut–Thyroid–Mitochondrial Interface

Micronutrients act as biochemical bridges between microbial metabolism, thyroid signalling, and mitochondrial function. Their availability helps determine the capacity of thyroid hormones to sustain oxidative metabolism during physical stress. In athletes, microbial integrity may influence both micronutrient absorption and turnover, making the gut–thyroid–nutrient interface an important contributor to recovery and allostatic stability [27,107].

#### 3.2.1. Functional Regulation of the Gut–Thyroid–Micronutrient Axis

The intestinal microbiota modulates luminal pH, bile acid metabolism, and redox balance, three interconnected factors that influence mineral solubility, nutrient absorption, and thyroid hormone activation.

Among these mechanisms, SCFA production plays a key role. SCFA-producing taxa such as *Faecalibacterium* and *Roseburia* acidify the intestinal lumen through fibre fermentation, a process that may enhance the solubility and bioavailability of iron, zinc, and iodine. They also contribute to epithelial integrity and have been linked, in experimental models, to increased activity of IAP and to the regulation of important mineral transporters such as NIS, DMT1, and ZIP4 [26,102,108]. These effects together may improve the availability of thyroid cofactors and favour effective endocrine–mitochondrial communication.

Additionally, microbial transformation of bile acids links lipid metabolism to thyroid regulation, suggesting a potential route to influence lipid oxidation, heat production, and rapid hormonal adaptation during physical exertion [35,100].

During sustained training, sweat loss, inflammation, and oxidative turnover increase micronutrient demand, while temporary dysbiosis can reduce absorption efficiency. The resulting functional deficiency may reflect adequate intake but diminished biological availability due to microbial or epithelial limitations [109].

When these microbial–endocrine mechanisms are preserved, mineral homeostasis, THs activation, and mitochondrial efficiency can be maintained, supporting flexible metabolic responses to physical stress and recovery demands in athletes.

#### 3.2.2. Functional Micronutrient Triads in Endocrine–Mitochondrial Regulation

Micronutrients can be organized conceptually into three functional axes that link microbial metabolism with endocrine and mitochondrial regulation (see Table 1) [16,110]:

##### Endocrine–Redox Regulation: Iodine and Selenium

Iodine provides the substrate for T_4_ and T_3_ synthesis. At the same time, selenium supports T_4_-to-T_3_ conversion and antioxidant defence through selenoproteins such as GPx, TrxR, and deiodinases (D1–3) [111].

The microbiota may influence their absorption and activation: *Lactobacillus* and *Bifidobacterium* species can enhance iodide solubility, and *Bacteroides* and *Clostridium* can reduce dietary selenate to bioavailable selenide, although evidence in humans remains limited [104]. In athletes, iodine loss via sweating and selenium-dependent enzyme turnover under oxidative stress suggest that this axis may be particularly relevant for maintaining thyroid function and redox protection during prolonged exertion [103,115].

##### Metabolic–Immune Efficiency: Iron and Zinc

Iron and zinc act as essential cofactors for thyroid peroxidase, cytochrome enzymes, and T_3_-dependent transcriptional processes [28,112].

Microbial metabolism can influence iron solubility and has been linked to the regulation of metal transporters in experimental studies, while inflammation-driven hepcidin elevations restrict systemic iron availability [116]. Similarly, zinc uptake relies on microbial regulation of ZIP4 transporters, which can be altered under dysbiosis and cytokine activation [113]. Deficiencies in either mineral may impair thyroid responsiveness and prolong recovery from training stress, reflecting the shared redox–immune link between metabolism and endocrine adaptation [20].

##### Immuno-Neuromuscular Integration: Vitamin D

Vitamin D, acting through its receptor (VDR), integrates endocrine, immune, and neuromuscular regulation. Experimental studies suggest that *Lactobacillus* and *Bifidobacterium* species may influence VDR expression and bile acid-dependent vitamin D activation, potentially modulating epithelial and immune homeostasis [27,114].

Adequate vitamin D levels supports calcium handling, muscle contractility, and thyroid–immune tolerance, whereas deficiency, which is common in indoor or low-sunlight athletes, has been associated with impaired mitochondrial function and prolonged recovery [110,117].

#### 3.2.3. Dysbiosis-Driven Deficiencies and Performance Drift

When dysbiosis persists, alterations in luminal pH, bile acid handling, and inflammatory tone may contribute to micronutrient malabsorption and redox stress. Reduced SCFA output has been linked, in experimental systems, to decreases in metal transporter activity, while elevations in IL-6 and hepcidin impair iron recycling and may alter selenium and zinc distribution [5,16]. These shifts could influence deiodinase function and antioxidant protection, lowering T_3_ availability and mitochondrial output, although these mechanisms have not been demonstrated directly in humans.

In the context of endurance training, these combined disturbances may manifest as slower recovery, reduced thermogenesis, and fatigue, features that conceptually resemble an energy-conservation phenotype within the broader model of maladaptive thyroid–microbiota interaction [20,118]. Conversely, restoring microbial balance through high-fibre, polyphenol-rich diets and targeted probiotics (e.g., *Lactobacillus plantarum 299v*) have been associated with improvements in mineral bioavailability and metabolic resilience, although their effects on thyroid–mitochondrial coupling in humans remain uncertain [27,119].

Altogether, micronutrients may operate as molecular translators between microbial activity and endocrine regulation. When available in balanced amounts, they help integrate gut-derived metabolic stimuli with thyroid signalling and mitochondrial function, contributing to the physiological substrate of performance resilience in athletes.

### 3.3. Integrative Model of the Gut–Thyroid–Mitochondrial Continuum in Athletes

#### 3.3.1. Phases of Adaptation and Overload

The continuum between adaptation and fatigue, driven by the interplay of microbial diversity, thyroid signalling, and mitochondrial function, can be represented through four functional stages integrating metabolic, redox, and endocrine responses (see Table 2).

The functional phenotypes summarised in Table 2 do not represent discrete clinical categories but mechanistic constellations derived from converging evidence in human athletes, clinical cohorts with altered thyroid function, and preclinical models of metabolic stress. Associations between endocrine profiles, microbial features, mitochondrial dynamics, and performance outputs are therefore interpretative and grounded in recurring patterns described across the literature [2,7,16,20,26,43,62,84]. Their purpose is to integrate dispersed findings into a coherent physiological framework rather than to propose diagnostic entities.

#### 3.3.2. Recovery, Resilience, and Endocrine Flexibility

Transitioning back from functional suppression to metabolic efficiency depends on restoring microbial diversity, micronutrient sufficiency, and thyroid–mitochondrial coupling. SCFA recovery reactivates AMPK–SIRT1 pathways and D1/D2 activity, while adequate selenium and zinc restore antioxidant defence and hormonal sensitivity [5,98]. This enables a return to the eubiotic, high-efficiency state characterized by optimal redox balance and flexible thyroid output.

Such resilience illustrates the allostatic principle of exercise endocrinology: stability through dynamic adaptation. Athletes with greater microbial diversity and micronutrient reserves demonstrate faster transitions across the continuum, preventing chronic energy deficiency and maintaining endocrine plasticity under load [2].

#### 3.3.3. Integrative Synthesis and Physiological Relevance

Collectively, the gut–thyroid–mitochondrial system operates as a multidimensional regulator of performance. Microbial metabolites shape thyroid activation and mitochondrial turnover; micronutrients provide biochemical substrates for enzyme function; and endocrine flexibility governs recovery kinetics. Disruption of any component propagates across the network, blurring the boundary between adaptation and maladaptation.

This integrative model provides the mechanistic framework for precision-based interventions that combine nutritional periodization, microbiota modulation, and micronutrient optimization.

## 4. Integrative and Translational Perspectives for Precision Exercise Physiology

### 4.1. Translating Mechanisms into Strategies

The mechanisms discussed in this review focus on a core physiological idea: thyroid function, microbial balance, and mitochondrial resilience operate together as an integrated system that controls energy production, redox balance, and adaptive recovery. Disruption of any part, due to dysbiosis, nutrient imbalance, or chronic stress, lowers allostatic flexibility and impairs performance capacity [121]. In contrast, restoring this balance requires coordinated interventions that rebuild microbial diversity, hormone synchronization, and mitochondrial stability.

In athletic settings, these molecular processes lead to measurable differences in fatigue resistance, recovery speed, and hormonal resilience. Athletes with balanced thyroid–microbiota interactions show quicker recovery of metabolic rate and lower inflammation after exercise. At the same time, prolonged dysbiosis or nutrient shortages delay recovery and increase the risk of overtraining [122]. Keeping thyroid–microbiota health intact is therefore key to maintaining performance under ongoing stress.

Translating these mechanisms into actionable strategies involves aligning nutrition, training, and recovery with the gut–thyroid axis. This alignment forms the foundation of precision performance physiology: coordinating nutrient intake, microbial modulation, and hormonal responsiveness to optimize adaptation. Precision nutrition provides a framework for integrating dietary composition, micronutrient adequacy, and circadian rhythm regulation to enhance thyroid sensitivity and microbial function [123]. When these factors are synchronized, metabolic efficiency, redox balance, and recovery potential are maximized.

From a translational perspective, this integration requires ongoing feedback between physiological monitoring and molecular biomarkers. Indicators such as the rT_3_/T_3_ ratio, SCFA output, and heart rate variability (HRV) can act as early markers of endocrine efficiency and recovery status, guiding personalized nutritional and training adjustments [7]. This approach connects molecular regulation with applied physiology, enabling real-time modulation of load and recovery.

### 4.2. Nutritional Modulation of the Gut–Thyroid Axis

Diet composition remains the primary and most influential modulator of the gut–thyroid axis. Nutritional interventions can directly alter microbial diversity, thyroid hormone activation, and systemic inflammation, ultimately affecting an athlete’s ability to adapt to training stress. Strong evidence supports that dietary patterns high in fermentable fibre, polyphenols, and omega-3 fatty acids influence microbial ecology and redox balance [32,124].

#### 4.2.1. Prebiotic Fibres and Microbial Resilience

Prebiotic fibres such as inulin, galacto-oligosaccharides (GOS), and fructo-oligosaccharides (FOS) promote beneficial bacteria (e.g., *Bifidobacterium*, *Lactobacillus*, *Faecalibacterium*) and increase SCFA production. These metabolites strengthen gut barrier function, stimulate IAP, and activate AMPK–SIRT1–PGC1α pathways, which connect gut metabolism to thyroid-dependent mitochondrial efficiency [32]. In athletes, regular fibre intake supports recovery and helps prevent low-T_3_ states during energy deficits or travel stress.

#### 4.2.2. Polyphenols and Anti-Inflammatory Signalling

Polyphenol-rich foods (berries, green tea, cocoa, extra-virgin olive oil) modulate NF-κB and Nrf2 pathways, boosting endogenous antioxidant defences and lowering pro-inflammatory cytokines like IL-6 and TNF-α [5]. These compounds also act as microbial substrates, promoting butyrate-producing species and improving bile acid composition. Through these mechanisms, they indirectly support thyroid hormone activation by reducing oxidative damage to deiodinases and enhancing systemic redox potential.

#### 4.2.3. Omega-3 Fatty Acids and Hormonal Stability

Omega-3 polyunsaturated fatty acids (EPA and DHA) further stabilize the gut–thyroid–immune axis by reducing TLR4 activation and increasing membrane fluidity in enterocytes and possibly in thyroid cells [125,126,127]. Emerging evidence also indicates that omega-3s influence the gut microbiota, promoting beneficial taxa such as *Akkermansia* and *Bifidobacterium*, improve SCFA levels, and thus support microbial–thyroid signalling [128]. In sports environments, adequate omega-3 intake supports recovery, boosts immune function, and helps prevent exercise-induced dysbiosis.

#### 4.2.4. Dietary Patterns and Long-Term Adaptation

Holistic dietary models such as the Mediterranean diet or well-balanced plant-based approaches include prebiotic fibre, antioxidant phytochemicals, and anti-inflammatory lipids within a balanced energy framework. These dietary patterns are consistently linked to greater microbial diversity, higher micronutrient density, and better metabolic efficiency, supporting systemic resilience and sustainable performance [129,130].

Adequate intake of essential micronutrients, including selenium, zinc, iodine, iron, and vitamin D, further enhances the metabolic and endocrine benefits of these dietary patterns. Each of these elements has a specific role in thyroid hormone synthesis and activation, redox balance, and immune regulation [85]. Selenium and zinc support antioxidant defences and deiodinase activity; iodine and iron are necessary for thyroid hormone biosynthesis; and vitamin D influences immune–endocrine communication and muscle recovery capacity [131].

In athletes, insufficient intake or poor bioavailability of these micronutrients can contribute to subclinical thyroid dysregulation, impaired energy metabolism, and fatigue. These changes are generally reversible with nutritional repletion and adequate energy intake [85]. Therefore, maintaining a balanced micronutrient status within a Mediterranean-like dietary framework is a practical strategy to sustain endocrine balance, optimize recovery, and promote long-term metabolic resilience in active populations.

#### 4.2.5. Applied Precision Strategies

Effective translation involves aligning dietary composition with training load and recovery cycles.

For endurance athletes, a fibre intake of about 40 g/day (inulin + GOS), at least 800 mg/day of polyphenols, and approximately 2 g/day of EPA + DHA boost SCFA production, improve T_3_/T_4_ balance, and support mitochondrial function [124].

For strength or power athletes, moderate fibre intake (25–30 g/day), approximately 1.5 g/day of omega-3 EPA + DHA, around 15 mg/day of zinc, and optimal vitamin D levels (40–60 ng/mL) support anabolic sensitivity and reduce inflammation. Supplementing with *Lactobacillus plantarum 299v* alongside iron-rich meals boosts ferritin levels without raising hepcidin, demonstrating precision co-supplementation [119].

Through these nutritional pathways, the gut–thyroid axis serves as a flexible interface that integrates microbial and hormonal signals to drive metabolic adjustments. In athletes, dietary quality and microbial diversity affect hormonal recovery and substrate use during and after training [32].

### 4.3. Exercise–Microbiota–Thyroid Triad

Beyond nutrition, physical training interacts with the gut–thyroid axis to regulate metabolic adaptation. Exercise changes microbial composition, influences endocrine sensitivity, and promotes mitochondrial turnover. These responses depend on training volume, intensity, and recovery status, which determine whether outcomes are beneficial or harmful. Moderate and consistent workloads support eubiosis and thyroid resilience, while chronic overload or insufficient recovery leads to dysbiosis, inflammation, and temporary thyroid suppression. Exercise-induced endocrine fluctuations such as acute TSH and fT_4_ increases with temporary T_3_ reduction, reflect adaptive recalibration of thyroid function under energetic stress [2,20,22].

#### 4.3.1. Microbial Responses to Exercise

Controlled training promotes beneficial taxa such as *Lactobacillus*, *Akkermansia muciniphila*, and *Faecalibacterium prausnitzii*, resulting in increased production of SCFA and greater microbial diversity [132]. In athletes, this SCFA-driven metabolic pathway is associated with improved endurance, faster lactate clearance, and enhanced redox recovery after high-intensity exercise [32].

#### 4.3.2. Inflammatory and Hormonal Balance

When training surpasses the body’s recovery capacity, microbial diversity tends to decrease, intestinal permeability increases, and circulating IL-6 and TNF-α levels elevate. These pro-inflammatory cytokines stimulate hepcidin production, reducing iron bioavailability and impairing erythropoiesis [115,133]. Elevated IL-6 and hepcidin are key mediators of the transient “inflammatory anemia” and low-energy signalling seen in overreached athletes.

Such changes can suppress the HPTaxis and reduce peripheral conversion of T_4_ to T_3_, resembling early endocrine features of NTIS [134,135]. These adaptive endocrine responses also overlap with the low-T_3_ phenotype observed in RED-S [136,137]. Overall, these findings suggest that inadequate recovery and low energy availability share common molecular pathways involving inflammation, iron regulation, and thyroid hormone metabolism.

#### 4.3.3. Thyroid-Driven Modulation of Exercise Metabolism

THs regulate mitochondrial oxygen consumption, carbohydrate oxidation, and contractile efficiency. During endurance activity, T_3_ increases glycolytic enzyme expression and mitochondrial respiration, while T_4_ acts as a reserve for local conversion. Exercise-induced changes in D2/D3 activity refine these hormonal responses to meet energy demands [66]. The microbiota helps maintain SCFA and bile acid pools that support peripheral T_4_ to T_3_ conversion and help preserve redox balance in muscle tissue [35,44].

#### 4.3.4. Adaptive Versus Maladaptive Response

When microbial or energetic support becomes insufficient, deiodinase activity decreases, and active T_3_ levels fall, in a coordinated downregulation that conserves ATP and reduces oxidative stress. Short-term, this serves as an adaptive fatigue threshold; however, if it persists, it can lead to maladaptive overtraining characterized by chronic inflammation and metabolic rigidity. Restoring microbial diversity, energy intake, and micronutrient levels reverses this state, normalizing T_3_ levels and enhancing performance output [57,138].

#### 4.3.5. Integrative Physiological Outcome

The exercise–microbiota–thyroid triad forms an allostatic network in which microbial, endocrine, and mitochondrial signals interact to sustain performance. Training enhances this triad when recovery and nutrition preserve microbial stability and mineral balance; otherwise, dysbiosis and inflammation can impair endocrine function. Recognizing these feedback mechanisms helps practitioners adjust load and recovery to maintain the endocrine–microbial balance essential for long-term adaptation. Biomarkers such as SCFA levels, rT_3_/T_3_ ratio, and HRV can serve as early indicators of this balance, guiding precise interventions to prevent maladaptive fatigue [7]. Maintaining a healthy microbiota supports faster mitochondrial recovery and thyroid reactivation after exertion [5,6].

### 4.4. Environmental and Nutritional Stressors

Environmental and nutritional stressors further disrupt the gut–thyroid–mitochondrial balance, influencing microbial diversity, hormone regulation, and metabolic functions. In athletes, these factors often interact, transforming adaptive metabolic flexibility into chronic fatigue, low-grade inflammation, or subclinical thyroid suppression. Their impact depends on exposure duration, recovery quality, and micronutrient levels.

#### 4.4.1. Thermal and Circadian Stress

Extreme temperatures, dehydration, and disrupted circadian rhythms can affect gut permeability and the microbial makeup. Heat exposure increases intestinal permeability and endotoxin translocation, triggering TLR4-mediated inflammation and temporary hypothyroidism [139]. Elevated cortisol and catecholamines suppress TSH release and decrease T_4_ to T_3_ conversion, impairing thermoregulation and endurance efficiency [20]. In contrast, cold exposure enhances brown adipose tissue thermogenesis via T_3_ activation but can deplete iodine and selenium reserves if prolonged [140]. Athletes with diverse gut microbiota and adequate iodine and selenium stores tend to maintain better thermoregulatory stability after exertional heat stress and recover thyroid-driven metabolism more quickly. Circadian disruption from late training, travel, or poor sleep reduces microbial rhythmicity and thyroid hormone oscillations [141]. Misalignment between feeding and hormonal cycles weakens metabolic coordination and hampers recovery.

#### 4.4.2. Nutritional Restriction and Energy Imbalance

A chronic caloric deficit or low-carbohydrate intake suppresses deiodinase activity and limits SCFA synthesis by decreasing the availability of fermentable substrates [20]. This dual suppression of the metabolic-microbial axis reduces active T_3_ levels and mitochondrial output, resembling NTIS. Deficiencies in selenium, iron, and zinc further amplify these effects by impairing deiodinase activity and antioxidant defences [16].

In endurance athletes, sustained low energy availability and repeated hepcidin elevations impair iron absorption and thyroid hormone metabolism, contributing to fatigue and reduced metabolic efficiency [115,135,137]. Hepcidin synthesis is stimulated by IL-6, particularly under low-carbohydrate or energy-restricted conditions [142,143], while iron deficiency itself can limit TPO activity and T_4_ to T_3_ conversion [112,144]. Strategic refeeding, adequate carbohydrate intake, and micronutrient monitoring are therefore essential to restore iron availability, normalize thyroid function, and optimize recovery.

#### 4.4.3. Pollutants and Endocrine Disruptors

Environmental contaminants, including bisphenols, phthalates, and heavy metals, act as endocrine disruptors, interfering with iodine uptake and thyroid receptor binding [27]. Many also alter microbial composition, enriching proinflammatory species and impairing detoxification pathways [145,146]. Persistent exposure increases oxidative stress, disrupts bile acid metabolism, and decreases T_3_ responsiveness in liver and muscle tissues. Nutritional antioxidants and fibre help counter these effects by binding xenobiotics and supporting microbial resilience.

#### 4.4.4. Integrated Physiological Impact

These environmental and nutritional stressors collectively weaken the adaptive capacity of the gut–thyroid–mitochondrial axis. During high training loads or insufficient recovery, the combined effects increase oxidative stress and suppress endocrine function, raising the risk of fatigue and infection. Recognizing and addressing these factors through hydration, antioxidant nutrition, circadian alignment, and controlled exposure maintains allostatic resilience and supports sustained performance. Monitoring markers like cortisol, rT_3_/T_3_ ratio, and SCFA output can help identify early signs of maladaptation and guide prompt intervention.

### 4.5. Gene–Microbiota Interactions

At the molecular level, genetic polymorphisms affect how thyroid and microbiota pathways respond to nutritional and environmental stressors. These variants influence key factors in energy production and recovery, such as deiodinase efficiency, receptor sensitivity, and vitamin D signalling. The phenotype that develops in each athlete is thus a combination of genetic potential and microbial influence, shaping individual resilience to training stress and metabolic imbalance [30,147].

#### 4.5.1. Deiodinase and Thyroid Receptor Polymorphisms

Variations in the DIO1 and DIO2 genes control the conversion of T_4_ to T_3_ and affect local hormone levels in metabolically active tissues. Reduced DIO2 activity limits mitochondrial biogenesis and oxidative capacity, which makes recovery slower and increases fatigue under energetic stress [16]. Similarly, polymorphisms in TSHR alter how the body responds to TSH signalling, impacting endocrine adaptation to caloric or training deficits. The gut microbiota influences these responses by affecting selenium and zinc availability, essential cofactors for deiodinase enzymes, and by regulating the inflammatory environment that influences gene expression. Long-term dysbiosis or inflammation can suppress DIO2 transcription, mimicking genetic downregulation, which can be reversed through microbial restoration [148].

#### 4.5.2. Vitamin D and Immunometabolic Regulation

Polymorphisms in the VDR gene, particularly the *FokI* and *TaqI* variants, impact both thyroid function and the composition of the microbiome. People with lower VDR activity produce fewer antimicrobial peptides, have weakened mucosal defences, and face an increased risk of autoimmune thyroiditis [27]. Adequate vitamin D levels enhance epithelial strength and microbial diversity, countering genetic predispositions to inflammation. These effects emphasize the dual role of VDR signalling as both an immunomodulator and a metabolic regulator that connects the gut–thyroid axis.

#### 4.5.3. FOXO3A, MTHFR, and Redox Control

FOXO3A and one-carbon genes such as MTHFR plausibly link redox control and methylation to thyroid–microbiota crosstalk. FOXO3A coordinates oxidative-stress defences and mitochondrial quality control relevant to endurance stress. At the same time, common MTHFR variants modulate homocysteine/one-carbon flux with documented associations to thyroid disorders in some cohorts/meta-analyses, but with contradictory results. Microbial metabolites such as SCFAs, microbe-derived folate, and B12, feed one-carbon/epigenetic pathways and intersect the gut–thyroid axis, supporting a bidirectional microbiome–host–gene interface. However, how it can affect the epigenetic mechanism of deiodinase remains emerging (Fasano et al., 2019; Liu et al., 2025; van Vliet et al., 2024; Yang et al., 2022) [149,150,151,152].

#### 4.5.4. Implications for Personalized Performance

Understanding gene–microbiota interactions supports a transition from generalized dietary recommendations to targeted precision strategies. For instance, individuals carrying the DIO2 Thr92Ala polymorphism, which shows altered enzyme activity and reduced T_3_ availability, may require optimized selenium intake given the enzyme’s selenoprotein nature. Although emerging clinical protocols are beginning to stratify selenium interventions by DIO2 genotype, genotype-specific trials in athletes remain lacking [153].

Similarly, VDR polymorphisms have been shown to modify physiological response to vitamin D supplementation [154,155,156], supporting the rationale for genotype-guided dosing to maintain immune–endocrine balance.

Integrating genomic, microbiome, and metabolic data thus provides a promising foundation for individualized nutritional strategies aimed at sustaining thyroid function, redox balance, and performance adaptation. Therefore, robust, genotype-stratified randomized trials are still required.

### 4.6. Predictive and Causal Models

Integrating the genetic, microbial, and endocrine layers of regulation requires predictive and causal models that connect molecular signals to physiological outcomes. Advances in multi-omic analysis and computational modelling now allow quantification of how genetic variants, microbial metabolites, and environmental stressors collectively influence thyroid function and performance adaptation. These tools turn descriptive associations into mechanistic insights, enabling real-time prediction of metabolic efficiency and recovery capacity [20,147].

#### 4.6.1. Multi-Omic Integration and Model Development

Multi-omic integration frameworks combine genomic, epigenetic, metagenomic, and metabolomic data to model the host–microbiota system as a unified adaptive network. Statistical learning algorithms such as elastic net regression, random forests, and gradient boosting can identify nonlinear relationships among microbial metabolites, including SCFA, bile acids, tryptophan derivatives, and thyroid-related biomarkers such as T_3_, T_4_, TSH, and rT_3_. Complementary causal-inference approaches, including Bayesian networks and structural equation modelling, help distinguish correlation from causation, clarifying the direction of gut–endocrine regulation [20,30].

Emerging multi-omics and wearable models are starting to predict performance decline, recovery patterns, and fatigue trends by integrating biochemical, microbial, and physiological data streams. Reduced microbiome diversity and increased levels of inflammatory cytokines have been linked to early performance decline in endurance settings [157], and theoretical frameworks suggest that altered thyroid hormone ratios, such as elevated rT_3_, could serve as common biomarkers of metabolic stress. Although direct multi-omic and sensor-based validation remains limited, these integrated models show promise as an early warning system for personalized recovery management [113].

#### 4.6.2. Personalized Prediction and Adaptive Monitoring

Predictive systems integrating diet, sleep, environmental, and biochemical data are emerging as powerful tools for forecasting thyroid function and overall metabolic resilience. Recent machine-learning frameworks continuously updated with metabolomic, hormonal, and microbiome data can act as digital physiological twins, simulating how individuals respond to nutritional or training changes [158,159]. Such adaptive models enable the early identification of responders and non-responders to dietary or micronutrient interventions, forming the computational basis for precision nutrition [160].

These multi-omic predictive pipelines are particularly relevant for endurance and high-intensity sports, where allostatic flexibility is continuously challenged. Integrating thyroid, microbial, and metabolic datasets allows coaches and clinicians to personalize recovery periods, optimize iron and selenium support, and adjust training loads with improved accuracy. In this context, machine-learning approaches have demonstrated diagnostic potential for classifying thyroid-related metabolic phenotypes [161], illustrating the convergence of systems biology and performance analytics.

#### 4.6.3. Causal Reasoning and Translational Application

Moving beyond predictive correlation involves causal reasoning frameworks that uncover the active factors driving endocrine and performance variability. By integrating genetic predispositions (DIO2, VDR, MTHFR) with microbial and metabolic data, causal models can pinpoint specific variables, such as selenium depletion or bile acid imbalance, that directly affect T_3_ activation or mitochondrial efficiency. This method enables in silico testing of targeted interventions (e.g., selenium repletion, probiotic modulation) before implementation, speeding the translation of molecular insights into practical applications [27,148].

Ultimately, these models enhance precision sports medicine, where continuous data integration supports endocrine balance, microbial health, and long-term performance. The framework links molecular biology, computational prediction, and applied physiology within a single, adaptive diagnostic system. Early detection of elevated rT_3_, decreased SCFA output, or shifts in microbial diversity has been successfully proposed as a pre-symptomatic indicator of overtraining and functional thyroid suppression in athletes. Monitoring rT_3_ and SCFA patterns allows early identification of allostatic overload before fatigue symptoms appear [85].

#### 4.6.4. Integrative Summary of Translational Strategies

The following table (Table 3) summarizes key interventions that influence the gut–thyroid–mitochondrial axis, arranged from foundational dietary measures to emerging predictive methods. These strategies incorporate nutritional, physiological, and technological domains, emphasizing modulatory rather than causal relationships.

Each intervention connects its main mechanistic pathway with microbial–endocrine effects and functional outcomes relevant to athletic performance, providing a clear translational overview of the evidence discussed throughout this section.

### 4.7. Integrative Framework for Personalized Performance

The integration of genetic, microbial, and endocrine data enables a shift from simple descriptive physiology to personalized performance science. In this framework, thyroid function, microbial ecology, and mitochondrial output are not isolated factors but parts of an interconnected system that manages energy efficiency, adaptation, and recovery. Instead of just maximizing output, the focus shifts to maintaining physiological coherence, in which thyroid activation, microbial diversity, and redox stability work together harmoniously [27,113].

Linking molecular signatures to physiological and performance outcomes enables an objective assessment of how diet, training, or environmental stress affects the gut–thyroid–mitochondrial axis. Multi-omic monitoring that combines genomics, metabolomics, and microbiome data could offer quantifiable biomarkers for evaluating allostatic load, endocrine flexibility, and recovery efficiency [30]. In athletes, early changes in SCFA production, microbial diversity, or rT_3_ trends serve as initial indicators of energy imbalance or excessive training stress [20]. Detecting these signatures enables timely intervention before functional suppression or performance decline occurs.

Precision nutrition can convert these molecular insights into practical, adaptable strategies tailored to the athlete’s microbial and hormonal profiles. Dietary composition, micronutrient support, and recovery methods are adjusted based on biological feedback, for instance, increased oxidative stress leading to altered selenium levels suggests antioxidant and trace element support. At the same time, shifts in microbial metabolites indicate the need for prebiotic or probiotic modifications [16]. This flexible approach replaces static guidelines with responsive adjustments, helping to maintain thyroid–microbiota–mitochondrial balance amid changing energy demands.

Ultimately, the goal is not just optimized performance but also sustainable endocrine and metabolic health. This holistic approach links molecular biology with applied sports medicine, making precision nutrition a vital part of adaptive athletic performance.

## 5. Conclusions

Thyroid allostasis provides a dynamic framework to understand how endocrine, nutritional, and microbial systems cooperate to sustain resilience under physiological stress. In athletes, this triad defines the capacity to maintain energy efficiency and recovery despite environmental, metabolic, or training-related perturbations.

The evidence analysed here indicates that micronutrient sufficiency, microbial diversity, and mitochondrial integrity form interdependent layers of thyroid regulation. Iodine, selenium, iron, zinc, and vitamin D remain essential cofactors for hormone synthesis, conversion, and signalling, yet their effectiveness depends on microbial modulation, barrier function, and systemic demand. Dysbiosis or chronic stress can disrupt these links, impairing both endocrine precision and metabolic flexibility.

Athletic adaptation illustrates this interconnection. Sweat and haemolytic losses, gastrointestinal strain, and oxidative turnover heighten vulnerability to micronutrient imbalance. Whether the outcome is enhanced resilience or chronic fatigue depends on the alignment of genetic predisposition, microbial ecology, and recovery dynamics. Integrative monitoring that combines endocrine, microbial, and metabolic markers may therefore improve early detection of maladaptation.

Future work should focus on longitudinal, multi-omic studies to validate causal pathways between microbiota composition, thyroid regulation, and performance. Machine-learning and causal-inference models offer promising tools for identifying predictive signatures and guiding individualized interventions.

Ultimately, thyroid allostasis should be regarded not as a fixed set point but as an adaptive capacity emerging from the endocrine–microbial–nutritional triad. Precision strategies that balance micronutrient intake, sustain microbial resilience, and respect recovery cycles may help preserve thyroid efficiency, prevent fatigue, and support long-term performance health.

## Figures and Tables

**Table 1:** Functional clustering of micronutrients within the gut–thyroid–performance axis.

Functional Group	Core Physiological Axis	Microbial Contribution	Performance Relevance	Key References
**Iodine (I) +** **Selenium (Se)**	**Endocrine–redox** **Regulation**	*Lactobacillus* and *Bifidobacterium* have been linked with increased iodide solubility; while *Bacteroides* and *Clostridium* can reduce dietary selenate/selenite to more bioavailable forms in experimental systems, potentially supporting deiodinase and antioxidant enzyme activity.	Contribute to thyroid hormone synthesis and activation (T_4_ → T_3_), and help maintain redox balance, which may influence fatigue and thermogenic responses during training	[16,27,111]
**Iron (Fe) +** **Zinc (Zn)**	**Metabolic–immune** **Efficiency**	SCFA-producing and lactate-producing taxa, such as *Lactobacillus plantarum* and *Faecalibacterium*, may enhance Fe^2+^ solubility and modulate the expression of metal transporters, such as DMT1 and ZIP4, in experimental models. At the same time, inflammation-driven increases in hepcidin can limit systemic availability.	Serve as cofactors for TPO, cytochrome enzymes, and THR structure, supporting oxygen transport, immune tolerance, and metabolic regulation under heavy training.	[20,112,113]
**Vitamin D**	**Immunoendocrine–** **neuromuscular** **integration**	Certain *Lactobacillus* and *Bifidobacterium* strains have been shown to influence bile acid signalling and VDR expression in experimental models, potentially modulating vitamin D activation and immune responses.	May support neuromuscular function, immune balance, and thyroid–immune tolerance, particularly in athletes with low sunlight exposure.	[27,110,114]

Simplified integration of micronutrient clusters within the gut–thyroid–mitochondrial framework. Each functional group may contribute to endocrine precision, metabolic efficiency, and physiological resilience under athletic stress. Microbial diversity support for the transformation, solubility, or absorption of several of these cofactors, although the magnitude of their effects in humans remains incompletely characterized.

**Table 2:** Adaptive and maladaptive phases of the gut–thyroid–mitochondrial continuum in athletes.

Stage	Primary Trigger	Dominant Mechanism	Micronutrient Status	Physiological Outcome	Recovery Potential	Key References
**1. Eubiosis and nutrient** **sufficiency**	Balanced training load; adequate intake	Nrf2/SIRT1–PGC1α ↑	Se, Fe, Zn, I ↑ SCFA ↑	Efficient thermogenesis full redox recovery	Full	[16,120]
**2. Early** **imbalance/** **mild deficiency**	Repeated load; marginal Deficit	IL-6/TNF-α ↑ D1/2 ↓; D3 ↑	Se/Zn ↓	Reversible T_3_ ↓ transient fatigue	High	[5,20]
**3. Redox–** **inflammatory coupling**	Overreaching; emerging dysbiosis	GPx/TrxR ↓ D1/2 ↓↓; D3 ↑↑	Se/Fe ↓ hepcidin ↑	Mitochondrial inefficiency energy conservation	Moderate	[118]
**4. Mitochondrial stress/** **immune drift**	Chronic dysbiosis and inflammation	Th1/Th17polarization Nrf2 ↓	Multi-cofactor depletion	Chronic fatigue thermogenesis ↓	Low	[2,27]

Sequential representation of the adaptive–maladaptive continuum linking microbial diversity, thyroid signalling, and mitochondrial performance in athletes. Stages 1–2 depict reversible physiological adaptations to training stress, whereas stages 3–4 reflect the transition toward chronic dysbiosis with cumulative endocrine and redox impairment. Micronutrient/cofactor status represents typical functional trends associated with each phase rather than fixed deficiencies [2,16,20,27,103,115].

**Table 3:** Translational interventions modulating the gut–thyroid–mitochondrial axis in athletes.

Intervention	Mechanistic Target/ Pathway	Microbial/ Endocrine Effect	Functional Outcome in Athletes	Level of Evidence/Cautionary Note	Key References
**1. Balanced diet** **rich in fermentable** **fibres and plant** **diversity**	Sustains SCFA production and mucosal integrity	Preserves microbial diversity and bile-acid metabolism	Maintains gut–thyroid equilibrium and supports recovery under training stress	Supported by observational and small interventional studies	[26,44,162,163,164,165]
**2. Prebiotic** **supplementation** **(inulin, GOS, FOS)**	↑ Butyrate → AMPK–SIRT1–PGC-1α signalling	Enhances deiodinase activity and intestinal barrier function	Associated with better recovery and reduced risk of low-T_3_ during energy deficit	Consistent mechanistic data; limited athlete-specific trials	[26,44,166,167,168,169]
**3. Polyphenol-rich foods (berries, cocoa, EVOO, green tea)**	Activation of Nrf2 and inhibition of NF-κB; secondary metabolism by *Bifidobacterium* & *Faecalibacterium*	Increases antioxidant enzymes (SOD, GPx); lowers IL-6 & TNF-α; supports SCFA and bile-acid homeostasis	May protect deiodinase function and redox balance during oxidative stress; supports mitochondrial efficiency and recovery	Mechanistic and human data are consistent; the magnitude depends on the matrix and microbiota profile	[5,170,171,172,173,174]
**4. Micronutrient sufficiency** **(Se, Zn, Fe, I, Vit D)**	Cofactors for DIO1/2, TPO, TrxR, VDR	Supports thyroid-hormone conversion and antioxidant defence	May prevent fatigue, anaemia, and redox imbalance under load	Strong biochemical evidence; monitor serum markers to avoid excess	[16,85,112,175]
**5. Synbiotic/** **probiotic protocols under adequate training load**	↑ *Faecalibacterium*, *Akkermansia*, *Lactobacillus* → ↑ SCFA, ↓ LPS	Modulates immune tolerance and DIO2 activity; stabilizes the intestinal barrier	May enhance thyroid efficiency and recovery when training and rest are balanced	Benefits are strain- and context-dependent; excessive load blunts response	[20,132,176]
**6. Omega-3 fatty** **acids (EPA, DHA)**	↓ TLR4 signalling; ↑ membrane fluidity	Improves T_3_ sensitivity in muscle and reduces IL-6	Associated with better recovery and reduced exercise-induced inflammation	Robust anti-inflammatory evidence; endocrine effects indirect	[103,177,178,179]
**7. Predictive multi-omic monitoring and digital twins**	Integration of genetic, microbial, and physiological data	Enables personalized tracking of thyroid–microbiota responses	Facilitates early detection of overtraining and adaptive nutrition adjustments	Emerging field; validation pending in athlete cohorts	[27,161]

## Data Availability

No new data were created or analysed in this study. Data sharing is not applicable to this article.

## Abbreviations

The following abbreviations are used in this manuscript:

AMPK	AMP-activated protein kinase
D1/D2/D3	Deiodinase types 1, 2, and 3 (protein level)
DIO1/DIO2/DIO3	Deiodinase isoforms 1, 2, and 3 (gene level)
ΔΨm	Mitochondrial membrane potential
FOXO3a	Forkhead box O3a
fT_3_	Free triiodothyronine
fT_4_	Free thyroxine
FXR	Farnesoid X receptor
GPx	Glutathione peroxidase
HPT axis	Hypothalamic–pituitary–thyroid axis
HRV	Heart rate variability
IL-6	Interleukin-6
LPS	Lipopolysaccharides
MCT8	Monocarboxylate transporter 8
MTHFR	Methylenetetrahydrofolate reductase
NAD^+^/NADH	Nicotinamide adenine dinucleotide (oxidized/reduced forms)
NRF1	Nuclear respiratory factor 1
NRF2	Nuclear respiratory factor 2
NTIS	Non-thyroidal illness syndrome
OXPHOS	Oxidative phosphorylation
PGC-1α	Peroxisome proliferator–activated receptor gamma coactivator 1-alpha
RED-S	Relative Energy Deficiency in Sport
ROS	Reactive oxygen species
rT_3_	Reverse triiodothyronine
rT_4_	Reverse thyroxine
SCFA	Short-chain fatty acids
SIRT1	Sirtuin-1
SOD2	Superoxide dismutase 2
T_2_	3,5-diiodo-L-thyronine
T_3_	Triiodothyronine
T_4_	Thyroxine
TGR5	Takeda G-protein-coupled receptor 5
THs	Thyroid hormones
THRs	Thyroid hormone receptors
TFAM	Mitochondrial transcription factor A
TLR4	Toll-like receptor 4
TMAO	Trimethylamine-N-oxide
TNF-α	Tumour necrosis factor alpha
TRH	Thyrotropin-releasing hormone
TSH	Thyroid-stimulating hormone
UCP2	Uncoupling protein 2
UCP3	Uncoupling protein 3
UCPs	Uncoupling proteins
VDR	Vitamin D receptor
